# Immobilization of the extracellular recombinant Lucky9 xylanase from *Bacillus subtilis* enhances activity at high temperature and pH

**DOI:** 10.1002/2211-5463.13010

**Published:** 2020-11-09

**Authors:** Sai‐sai Ding, Jin‐peng Zhu, Yang Wang, Bin Wu, Zongpei Zhao

**Affiliations:** ^1^ School of Grain Science and Technology Jiangsu University of Science and Technology Zhenjiang China; ^2^ College of Biotechnology and Pharmaceutical Engineering Nanjing Tech University Nanjing China

**Keywords:** alkali tolerance, biobleaching, immobilization, sodium alginate, thermostable, xylanase

## Abstract

In the paper industry, chlorine is often used to treat the pulp for bleaching. After pulping, a large amount of xylan is present in the fiber. Xylanase can be used to degrade xylan in an eco‐friendly process called biobleaching, which can help minimize the usage of chlorine in the delignification process. However, a bottleneck in the adoption of biobleaching is the cost of xylanase and the requirement that xylanase be active and stable at extreme conditions. Here, we investigated whether using sodium alginate beads to immobilize an extracellular xylanase from *Bacillus subtilis* (Lucky9) can reduce the potential cost of enzyme usage. The optimal pH and the activity of the immobilized enzyme were increased at optimal temperature compared with the free enzyme. In addition, immobilized xylanase was shown to be more stable than free xylanase. The results of this study suggest that the immobilized xylanase has potential applications in the biobleaching industry.

## Graphical abstract

Sodium alginate beads were used as a carrier to immobilize a previously reported extracellular xylanase from *Bacillus subtilis* Lucky9 expressed in *Escherichia coli*. The optimal pH and the activity of the immobilized enzyme were increased at optimal temperature compared with the free enzyme. Our results suggest that the immobilized xylanase has potential applications in the biobleaching industry.

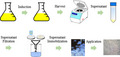

**Abbreviation**
DNS: 3,5‐dinitrosalicylic acid

Hemicelluloses are the second most abundant plant fraction available in nature [1], with the hemicellulose xylan accounting for as much as 30% of the dry weight of some plant tissues [2]. Xylan biodegradation requires the activities of several enzymes, among which xylanases (1,4‐beta‐D‐xylan xylanohydrolase, EC 3.2.1.8) play a key role [3]. In recent years, an increasing number of studies have investigated the use of microbial xylanases in the food and beverage industries and animal feeding industry [4, 5].

Due to their important industrial applications, many xylanase genes have been identified in bacteria and fungi [6]. Xylanases are grouped into glycosidase families based on the primary structure of the catalytic domains and are typically reported in glycosidase families 10 and 11 [7, 8].

In general, the use of free xylanase is often hampered by its lack of reusability, high production costs, low enzymatic activity and high consumption. However, immobilized enzymes can be reused in multiple cycles to reduce the production costs and overcome such technical bottlenecks [9, 10].

Because of its low cost, ease of use and nontoxicity, sodium alginate is one of the best candidates for use as carrier [11, 12, 13, 14]. A method for the covalent immobilization of xylanase to the surface of alginate beads has been previously described [15, 16, 17]. In this study, we used sodium alginate beads as carriers to immobilize a previously reported extracellular xylanase from *Bacillus subtilis* Lucky9 expressed in *Escherichia coli*. The characteristics of the immobilized enzyme were analyzed, and the results showed that it may have potential for use in the biobleaching industry [18].

In the paper industry, chlorine is often used to treat the pulp for bleaching. The primary role of chlorine in bleaching is to convert the residual lignin in the pulp into water or alkali‐soluble products [19]. During the kraft pulp process, part of the xylan is transferred to the fiber surface. After the pulping process, a large amount of xylan is present in the fiber. To this end, xylanase can be used to treat this xylan in an eco‐friendly process called biobleaching [20, 21], because xylanase pretreatment of kraft pulp can help minimize the usage of chlorine in the delignification process. However, a bottleneck in the adoption of biobleaching is that xylanase should be stable and active at extreme conditions, and the cost of the enzymes is high [22, 23, 24, 25, 26]. In this study, we used sodium alginate to immobilize xylanase, which may potentially reduce the cost of enzyme usage.

## Materials and methods

### Chemicals

Beechwood xylan, 3,5‐dinitrosalicylic acid (DNS) and d‐xylose were purchased from Yuanye Ltd. (Shanghai, China). All other chemicals and solvents used in this study were of analytical grade and obtained from Aladdin Ltd. (Shanghai, China).

### Xylanase expression and collection

**Fig. 1:**
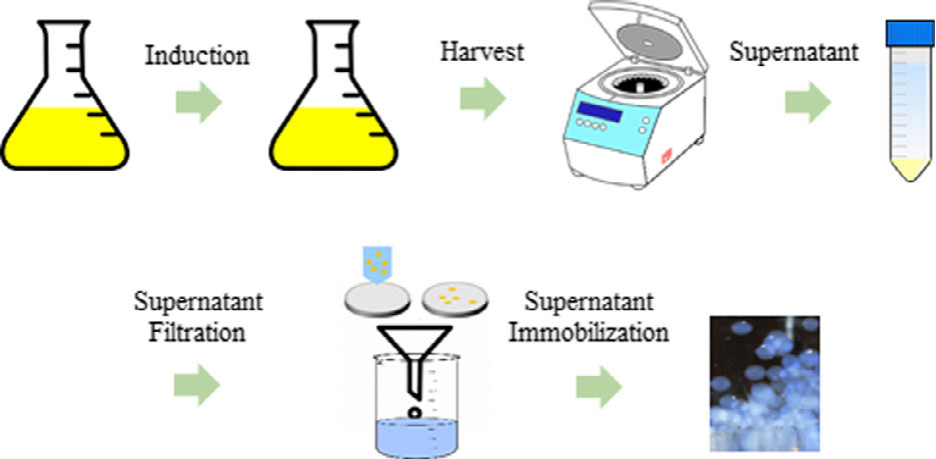
Schematic illustration of the process of expression and immobilization of recombinant xylanase from *Bacillus subtilis* Lucky9.

*E. coli* BL21‐DE3 was used for xylanase overexpression. Cells containing the plasmid of interest were cultured overnight in LB broth containing ampicillin (100 µg·mL^−1^). The cells were induced by adding 2 µL of a 0.1 m stock solution of IPTG (final concentration, 0.002 × 10^−3^ m) and further incubated at 20 °C overnight. After induction, the cultures were centrifuged (6000 ***g***; 10 min; 4 °C), and the supernatants were collected and filtrated using 0.45‐µm filters (Fig. 1).

### Sodium alginate bead preparation

The sodium alginate beads were produced by dropping a 2% (w/v) sodium alginate solution through a syringe into a 0.02 m CaCl_2_ solution as previously reported [10]. The ratio between the alginate and the CaCl_2_ solution was 1 : 2 (v/v). The beads were subsequently stored at 4 °C overnight to allow them to harden.

The alginate beads were further activated by dipping them into a 2% (w/v) glutaraldehyde solution. The activation process was performed at room temperature with orbital stirring for 4 h using a 1 : 10 ratio of beads: glutaraldehyde solution at 400 r.p.m. [10]. After activation, the beads were thoroughly washed to remove any unbound glutaraldehyde, after which they were ready for use.

### Xylanase immobilization

The filtrated culture was divided into 100 aliquots to calculate the amount of immobilized xylanase required for further use. The aliquots were mixed with the activated beads at a ratio of 1 : 1 (v/w). The enzyme and bead mixture was then mixed with shaking at 200 r.p.m. for 2 h using an orbital shaker. After activation, the beads were thoroughly washed with distilled water.

### Activity assay and protein determination of xylanase

Xylanase activity was measured using the method of Khanna and Gauri [27]. The beechwood xylan solution (1%) and the enzyme preparation were performed at 50 °C for 10 min. The standard reaction mixture contained 100 µL of dissolved xylan substrates with 50 × 10^−3^ m sodium acetate buffer (pH 5), 100 µL of enzymes/beads and 800 µL of buffer (50 × 10^−3^ m sodium acetate). The reaction was stopped by adding 1000 µL of DNS reagent and boiling for 30 min. Subsequently, the reaction mixture was measured at 540 nm.

One unit of xylanase was defined as the amount of enzyme required to release 1 mol reducing sugar as xylose equivalent per minute under the assay conditions. All the experiments were performed in triplicate, and the results are expressed as the means ± SD.

Protein concentration was determined using BSA as a standard. The protein content of the crude and immobilized enzyme fractions was measured by monitoring the optical density at 280 nm.

### Effects of pH and temperature on the activity and stability of free and immobilized xylanase

The activity and stability of immobilized xylanase were assayed at different temperatures and pH values using the enzyme assay described earlier. The effect of temperature on the activity and stability of xylanase was evaluated over a temperature range of 30–80 °C (pH 5). The effects of pH on the activity and stability of free and immobilized xylanase were investigated at different pH values: sodium acetate buffer (pH 3.0–6.0), sodium phosphate buffer (pH 6.0–8.0) and Tris–HCl buffer (pH 8.0–10.0) at 50 °C.

### Effect of xylanase on waste paper pulp

The paper pulp was prepared according to a previous study [28]. Immobilized xylanase‐treated pulp was prepared by treating 5% (w/v) pulp (pH 8) with an enzyme dose of 20–100 U·mL^−1^ per gram of pulp for 1 h at 50 °C with gentle shaking. Then, the treated pulp was filtered, thoroughly washed and dried at 50 °C for 3 h. A control was added without enzyme treatment. The Kappa number was used to evaluate the efficiency of the treatment process (TAPPI 1990). The brightness of the pulp was calculated using the method of Jordan and Popson [29]. The reducing sugar content in supernatant was measured by the DNS method, and chromophores and hydrophobic compounds were measured at 237 and 465 nm, respectively [30].

The immobilized enzyme was washed with distilled water and then added to a fresh reaction solution to start a new enzymatic reaction cycle to assess reusability. Experiments were repeated for eight cycles. The highest activity measured under the corresponding temperature or pH ranges was designated as 100%, and the activities at all the remaining temperatures and pH values were proportional to the highest activity.

## Results

Recombinant xylanases containing pelB signal peptides have been previously shown to be secreted into the culture medium [18]. A previous study showed that the recombinant enzyme was extracellularly expressed and based on this characteristic, we designed an immobilization method to use sodium alginate beads as carrier to immobilize the xylanase.

### Optimum pH and temperature

**Fig. 2:**
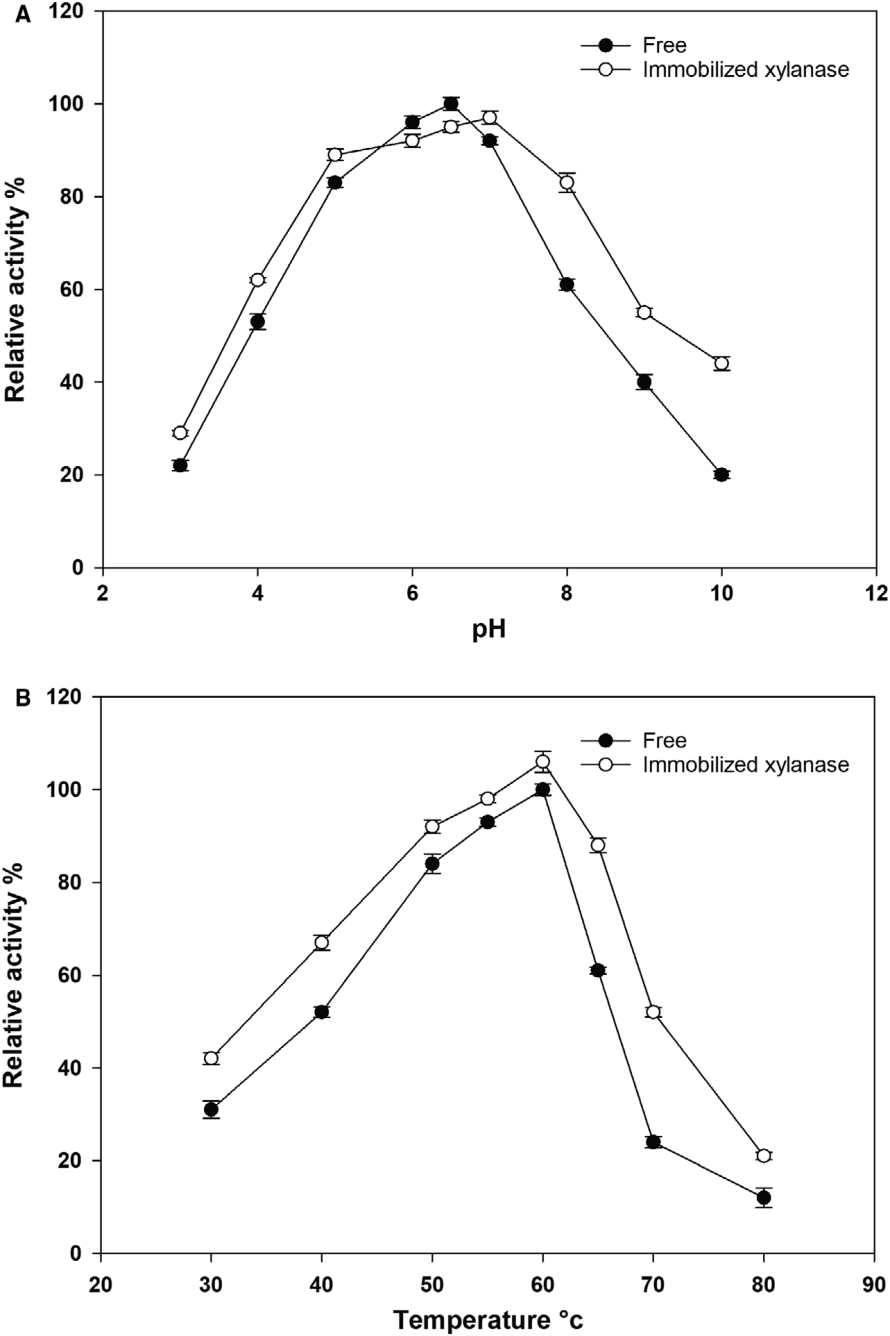
Influence of pH and temperature on the activity of the recombinant xylanase from *Bacillus subtilis* Lucky9. (A) Effect of temperature on recombinant xylanase activity (activity is relative to the free enzyme at pH 6.5 and 60 °C). (B) Effect of pH on recombinant xylanase activity (activity is relative to the free enzyme at pH 6.5 and 60 °C). Each value represents the mean of triplicate measurements and varied from the mean by not more than 15%; error bars indicate the standard deviation.

To determine the characteristics of the immobilized xylanase, we tested the free/immobilized xylanase under different pH and temperature conditions. Our results suggested that the optimal pH of the immobilized xylanase shifted to pH 7 and had 5% higher activity (430 U·mL^−1^) than that of free xylanase (Fig. 2). At alkaline pH values, the immobilized xylanase exhibited better activity than the free enzyme, suggesting that the immobilization enhanced the tolerance of the enzyme to alkaline conditions. This characteristic was also observed over an acidic pH range, where the immobilized xylanase had more than 10% higher activity than free xylanase.

In addition, the optimal temperature was also tested, with the results indicating that the optimal temperature of both the free and immobilized enzyme was 60 °C, where the immobilized xylanase showed 5% higher activity (495 U·mL^−1^) than free xylanase (Fig. 2). However, at 65 °C, the immobilized xylanase showed better activity, ~1.5 times higher than the free enzyme. At 70 °C, the activity of both xylanase forms dramatically decreased, although the immobilized xylanase still showed ~2 times higher activity than the free xylanase.

### Thermostability and reusability

**Fig. 3:**
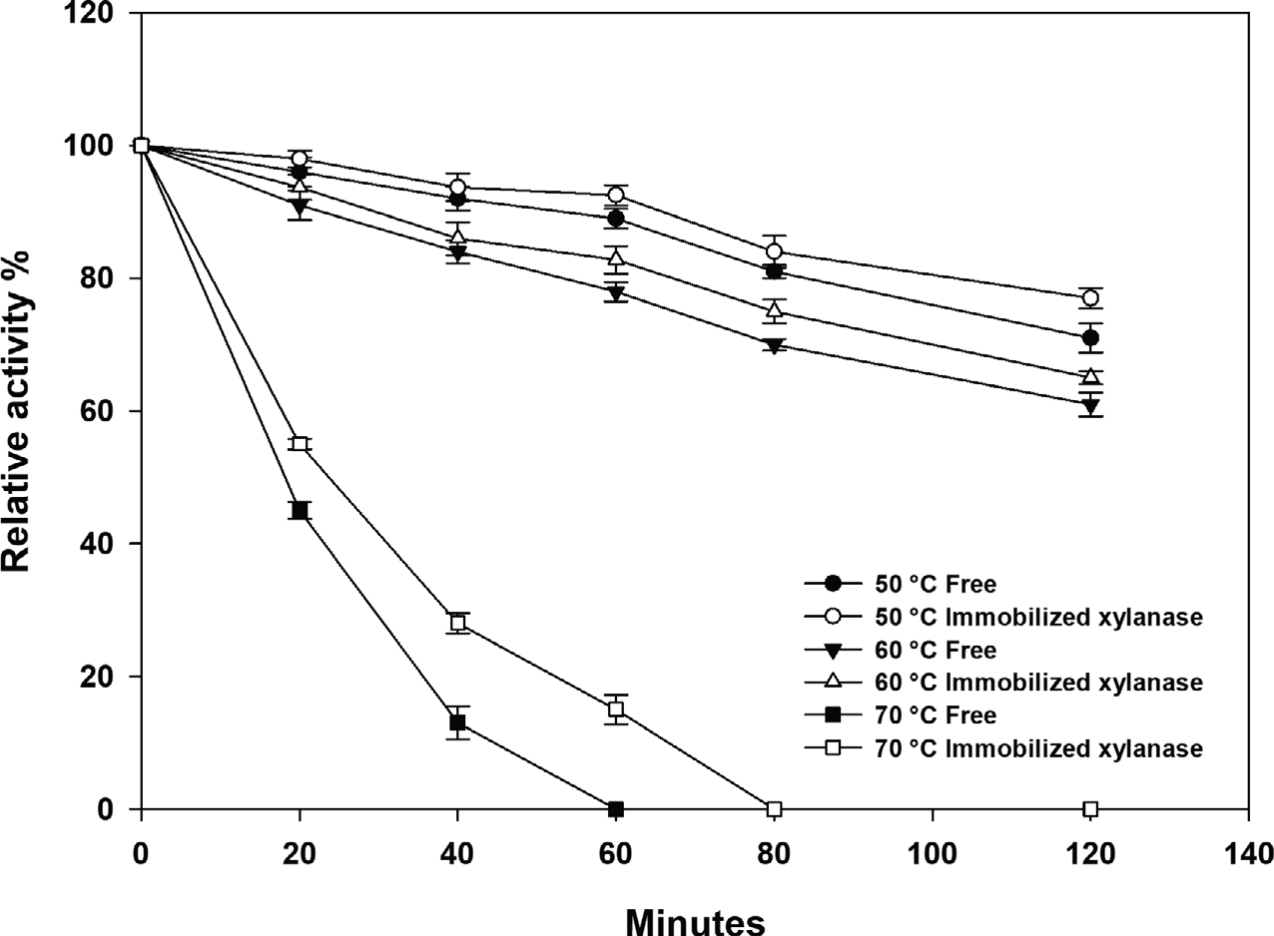
Relative activity of the recombinant xylanase from *Bacillus subtilis* Lucky9 in the stability assay. The activity is relative to that of the immobilized xylanase at 50 °C, 60 °C and 70 °C, respectively. Each value represents the mean of triplicate measurements and varied from the mean by not more than 15%; error bars indicate the standard deviation.

The optimum temperature analysis showed that immobilized xylanase exhibited better tolerance at higher temperatures, suggesting that its thermostability would also be improved. We assayed the free and immobilized enzymes at 50–70 °C, and the results showed that the immobilized xylanase had greater thermostability at 70 °C than the free enzyme, lasting for 20 min longer (Fig. 3). From 50 °C to 60 °C, the thermostability of the immobilized xylanase improved, with a relative activity that was 2–5% higher than that of free xylanase.

### Application in pulp biobleaching

After bioleaching, immobilized xylanase‐treated pulp samples were studied, and the Kappa number and brightness were obtained. After the immobilized xylanase treatment, a decrease in the Kappa number and an increase in brightness were observed, suggesting that lignin was released. In addition, compared with the untreated example (Table 1), the Kappa numbers observed using various amounts of immobilized xylanase were reduced, and the brightness was improved, which proved the catalytic efficiency in the reaction. Increased chromophore and hydrophobic compound contents were also observed, suggesting that immobilized xylanase efficiently removed these compounds (Table 1). Reducing sugar levels were also observed to increase, confirming that the pulp fibers were broken down and sugar was released when compared with the untreated sample.

**Table 1:** s paper pulp.

Parameter	Kappa number	Brightness (ISO units)	Chromophoric compounds (λ 237)	Hydrophobic compounds (λ 465)	Reducing sugar (mg·g^−1^)
Untreated	25.6	38.2	0.256	0.432	1.78
Xylanase (20 U·g^−1^)	22.1	39.6	0.981	0.755	2.64
Xylanase (40 U·g^−1^)	19.2	40.9	1.878	0.989	7.88
Xylanase (80 U·g^−1^)	17.8	41.7	2.231	1.652	10.25
Xylanase (100 U·g^−1^)	16.1	42.5	2.51	1.925	13.56

**Fig. 4:**
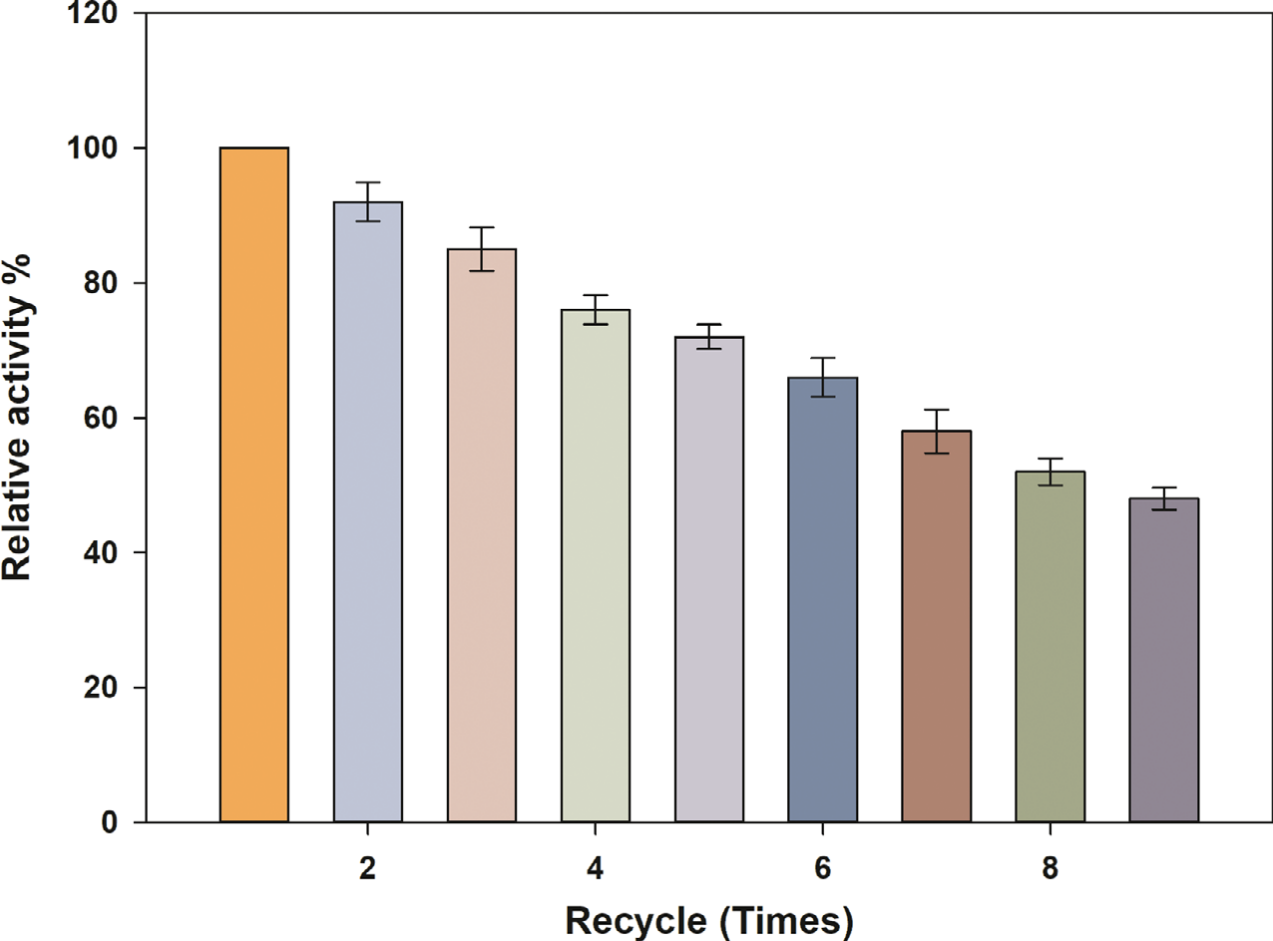
The reusability of the immobilized recombinant xylanase from *Bacillus subtilis* Lucky9. The activity is relative to the immobilized enzyme at pH 8 and 50 °C. Each value represents the mean of triplicate measurements and varied from the mean by not more than 15%; error bars indicate the standard deviation.

The reusability of immobilized xylanase was also tested, and the results suggested that after eight cycles, the immobilized xylanase retained ~50% activity (Fig. 4).

## Discussion

In a previous study, the xylanase used in this study was designed to be extracellularly secreted and was characterized as an alkali‐tolerant xylanase. Therefore, in this study, we assessed the potential of this xylanase for use in the paper industry, because xylanase‐aided bleaching of kraft pulps can reduce the consumption of chlorine chemicals in the bleaching process. However, to reduce the work of concentrating the xylanase from the culture, we designed an immobilization method using sodium alginate beads. Sodium alginate beads are relatively large, eco‐friendly, low in cost, have a high loading capacity and are easily prepared and widely used in immobilization studies.

In our study, we characterized the immobilized xylanase at various pH values and temperatures. The results indicated that the immobilized xylanase retained more than 90% of its activity at pH 6.5, and the optimal pH was shifted to pH 7 when compared with free xylanase. The immobilized xylanase showed more alkali tolerance than free xylanase. In the optimum temperature study, the immobilized xylanase showed better activity at 60 °C, and even at 70 °C showed greater stability than the free xylanase. The stability test results also confirmed that the immobilized xylanase is more stable. The reason may be that the covalent bond formation between glutaraldehyde and xylanase strengthens the structure, further promoting its tolerance under extreme conditions.

To test our idea, we used immobilized xylanase to pretreat kraft pulp. In the pulp pretreatment test, immobilized xylanase increased reducing sugar release and improved the brightness of the pulp. The reusability results also further confirmed the stability of the immobilized enzyme. Above all, our study proved the potential application of the immobilized xylanase in the kraft pulp biobleaching industry.

## Conclusion

In this study, a xylanase from *B. subtilis* Lucky9 expressed in *E. coli* was immobilized on sodium alginate beads. The results of this study suggested that immobilized xylanase can reduce the work of concentrating the enzyme and is more stable than free xylanase. Moreover, the immobilized xylanase exhibited higher activity at pH 7 than the free enzyme. The immobilized xylanase was applied in a biobleaching pretreatment study and confirmed the possibility of its use in the pulp industry. The results of our study offer insights into the potential use of immobilized xylanase in the paper industry.

## Conflict of interest

The authors declare no conflict of interest.

## Author contributions

JZ, YW and SD performed experimental studies and prepared the manuscript. BW prepared the seed culture and provided guidance for expression. ZZ coordinated the writing and outlined and revised the manuscript.

## Data Availability

Data can be available from the corresponding author upon reasonable request.
